# Clinical Characteristics and Cardiac Magnetic Resonance Findings in Patients With Hypertrophic Cardiomyopathy in Brunei Darussalam

**DOI:** 10.7759/cureus.86303

**Published:** 2025-06-18

**Authors:** Kah Cheong Tong, Nazar Luqman Bilgrami

**Affiliations:** 1 Acute Medical Unit, Stepping Hill Hospital, Manchester, GBR; 2 Cardiology, Heart of Melbourne, Melbourne, AUS; 3 Cardiology, Advara Heart Care, Melbourne, AUS

**Keywords:** brunei, brunei darussalam, cardiac magnetic resonance, clinical characteristics, hypertrophic cardiomyopathy, sudden death

## Abstract

Background

Hypertrophic cardiomyopathy (HCM) is a genetic heart disease that poses a risk for sudden cardiac death. It has not been studied systematically in Southeast Asian countries. The purpose of this study was to investigate the characteristics and patterns of fibrosis in HCM, as well as to determine the risk of sudden cardiac death in relation to age, sex, and myocardial fibrosis in Brunei Darussalam.

Methods

All patients diagnosed with HCM from 2011 to 2017 were studied in Brunei Darussalam. Data was acquired from clinical notes of these patients and studied for the demographic profile of the disease, clinical manifestations, and risks for sudden cardiac death.

Results

A total of 39 patients with HCM were identified, with a mean age at diagnosis of 47.46±12.27 years. Roughly half of HCM patients (51.3%) were found to be asymptomatic. A total of 30 patients underwent cardiac magnetic resonance (CMR). Myocardial fibrosis was found in 53.8%. This was more prevalent and significant in apical HCM compared to asymmetrical septal HCM, in 10 (83.3%) and nine (60%) patients (p=0.032), respectively. Apical and asymmetrical septal HCM were the most common types of HCM in the population, 15 (38.5%) and 16 (41%), respectively. Asymmetrical septal HCM presented with more murmurs than apical HCM patients, nine (56.3%) and one (6.7%) patients (p=0.001), respectively. Asymmetrical septal HCM has a higher median risk score compared to apical HCM, with medians of 2.05 (IRQ: 1.78) and 1.44 (IRQ: 0.85; p=0.025), respectively. There was no statistical difference in terms of clinical and imaging characteristics between male and female HCM patients.

Conclusion

Demographics and clinical presentation of the disease are comparable to those of other studies. However, the risk for sudden cardiac death was higher in asymmetrical septal HCM compared to apical HCM. We also found that myocardial fibrosis on CMR is more significant in apical HCM.

## Introduction

Hypertrophic cardiomyopathy (HCM) is a genetic heart condition that causes hypertrophy of the cardiac muscles of the left ventricle. It has been estimated to have a prevalence of 1 in 500 in the general population [1]. Severity of disease ranges greatly from asymptomatic to sudden cardiac death (SCD). It is the most common cause of SCD in young athletes, particularly due to intensive exercise [2]. Fifty-six percent of HCM patients present with symptoms such as palpitations, chest pain, syncope, dizziness and shortness of breath while the remaining forty-four percent were asymptomatic and remained unaware until incidental findings by electrocardiogram (ECG), echocardiogram, screening based on family history and clinical examination according to Eriksson et al. [3]. The criteria for diagnosis of HCM are defined by the presence of left ventricular thickening of 15 mm and above with the aid of echocardiography, especially associated with risk factors such as family history of HCM and sudden death, and syncope [4]. A significant outcome of the disease is SCD, which is difficult to diagnose if the patient is asymptomatic. Myocardial fibrosis is a pathological hallmark feature of HCM, which plays a role in causing malignant ventricular arrhythmias. This is one of the mechanisms for SCD in HCM patients [5,6]. Physical examination is often unremarkable, with no murmurs detectable. HCM may be suspected with the use of an ECG, but it is not specific to the condition [7]. Common tools used to screen for HCM are echocardiogram and cardiac magnetic resonance (CMR). The morphological characteristics and patterns of fibrosis in HCM can be studied to help assess the severity of the condition and classify the type of HCM with these tools [8].

HCM can be classified into several types based on its morphological features. The most common variant of HCM is septal HCM [9]. In septal HCM, hypertrophy of the septal wall divides the left chamber from the right chamber of the heart, while apical HCM presents with hypertrophy at the apical region of the heart. In concentric HCM, hypertrophy is not specified to a particular region. Other uncommon variants include focal HCM and HCM with right ventricular involvement [9].

There have been several studies describing the prevalence of types of HCM. Septal hypertrophy alone is the most common form of HCM, according to a study conducted in 2011 at Hamad General Hospital in Qatar [7]. In another study conducted in Taiwan, observation of septal hypertrophy was the most common variant of HCM in both men and women [10]. A study in 2003 suggested that the Apical form of hypertrophic cardiomyopathy is more prevalent in Japan [11]. The prevalence of the types of HCM is different in various countries. There is no previously available data on HCM patients in Brunei, nor has any research been conducted in this field. The purpose of this study is to investigate the clinical characteristics of myocardial hypertrophy and patterns of fibrosis in HCM, prevalence of different types of HCM, common clinical signs and symptoms and risks for hypertrophic cardiomyopathy as well as determining the risk of sudden cardiac death through sudden death risk score, in relation to age, sex and myocardial fibrosis in Brunei Darussalam.

## Materials and methods

Study design and subjects

This is a retrospective study of all patients diagnosed with hypertrophic cardiomyopathy since the start of the specialised clinic in 2011 to January 2017, at Raja Isteri Pengiran Anak Saleha (RIPAS) Hospital, Brunei Darussalam. Data was collected from December 2016 to January 2017. This study was approved by the Ethics Committee of RIPAS Hospital and Universiti Brunei Darussalam.

A total of 45 patients were identified from the cardiology department's file registry. After exclusions, a total of 39 patients diagnosed with HCM were identified. Patients were excluded if they were under 18 years of age, had other cardiac conditions such as hypertensive-induced hypertrophy, were pregnant, or had known infiltrative or storage diseases such as amyloidosis or Fabry disease. Data collected from each case included symptoms (palpitations, dizziness, syncope, shortness of breath, chest pain) at first presentation, family history, treatment received, and results of ECG, Holter's monitoring, echocardiography, CMR, and electrophysiology testing. All data was extracted from BruHIMS, the hospital's database, by supervisors with validated access. Information was combined in a data collection form that included the essential information as seen in Table 1, followed by compilation in Microsoft Excel. Any missing data during data collection was excluded from the analysis. Electrophysiology (EP) testing was not included in data analysis, as this was not routinely done, only in a few selected cases for further investigation of arrhythmia and symptoms. 

**Table 1:** Data collected for the study

Data category	Data
Demographic information	Age, sex, presence of hypertension, diabetes mellitus
Symptoms at first presentation	Palpitations, dizziness, syncope, shortness of breath, chest pain
Family history	Hypertrophic cardiomyopathy, sudden cardiac death
Treatment received	Automated implantable cardioverter defibrillator, beta-blockers, amiodarone, calcium blockers
Electrocardiogram	Left ventricular hypertrophy, ST-T changes
Holter's monitoring	Presence of ventricular tachycardia
Echocardiography	Hypertrophy, systolic anterior motion of the mitral valve, left ventricular obstruction, mitral regurgitation
Cardiac magnetic resonance	Anatomy and assessment of fibrosis
Electrophysiology testing	Ventricular tachycardia stimulation study

Diagnosis of HCM

The diagnosis of HCM was characterized by wall thickness of 15 mm or more without a definitive cause, on echocardiogram and/or cardiac magnetic resonance. If the wall thickening was 13 to 14 mm, then it required further evaluation of other factors such as family history and symptoms [12]. The Sokolow-Lyon criteria (SV1+RV5>3.5 mV) were used to define left ventricular hypertrophy (LVH) [13]. ST-T abnormalities were also noted. Types of HCM (apical, asymmetrical septal, concentric, focal) were characterized by wall thickening in regions of the heart by use of echocardiogram and/or CMR. In case of discrepancy of findings between echocardiogram and CMR, the latter was considered confirmatory. Late gadolinium enhancement on CMR was used to define the presence and severity of fibrosis as mild (1% to 25%), moderate (26% to 50%), and severe (more than 50%) [14]. CMR was performed either using a 1.5 Tesla or 3 Tesla scanner with an inversion time ranging from 300 ms to 450 ms. CMR used standard cine-segmented steady-state free precession (SSFP) sequences with a field of view (FOV) of 340 mm and a base resolution of 256. Myocardium tissue characterisation was assessed with short tau inversion recovery (STIR), fast low-angle shot 3D (FL3D), T1 and T2 mapping with the use of flow study if required, and fibrosis was assessed with high-resolution phase-sensitive inversion recovery (PSIR). The timing for LGE was acquired 10 minutes after intravenous contrast administration. Fibrosis is identified with thresholds greater than five standard deviations (SD). Other features such as mitral regurgitation, systolic anterior motion of the mitral valve, and intracavity obstruction were also looked at from the echocardiogram and CMR. Twenty-four-hour Holder ECG recordings were analysed for ventricular tachycardia, which is defined as three or more consecutive ventricular complexes at a heart rate of more than 100 beats per minute [15]. Generally, patients had a single 24-hour Holter, and if needed, multiple repeat recordings were allowed to look for ventricular tachycardia. EP testing results were noted as positive or negative. EP testing was done at 400 ms and 600 ms driving cycle length with up to three extra stimuli and burst pacing with and without Isuprel™ from right ventricular apex and right ventricular outflow tract [16].

The risk of sudden death in the next five years was estimated with the sudden death risk calculator, approved by the European Society of Cardiology [12]. The risk score calculator calculates the risk score by taking the patient's age, maximum left ventricular wall thickness, left atrial size, maximum left ventricular outflow gradient, family history of sudden cardiac death, non-sustained ventricular tachycardia, and syncope into account. Risk score groups were categorised into three categories: low risk (<4%), intermediate risk (4%≤x<6%), and high risk (≥6%) [12].

Data analysis

Statistical Package for Social Sciences (SPSS) version 23.0 (IBM, Inc., Armonk, US) was used to perform statistical analysis. Patient data was displayed as mean±standard deviation. Median and interquartile range were used when the data were not normally distributed. The Mann-Whitney test was used to replace the independent T-test if the assumptions were not met. Pearson's chi-squared test was used to compare two categorical variables. Fisher's exact test was used when the assumptions of the chi-squared test were not met. For all statistical tests, a p-value of less than 0.05 (p<0.05) was considered significantly different. The normal distribution of data was visually determined using histograms. Based on the assessment of normality, parametric or non-parametric tests were appropriately applied. Data skewness, if relevant, is presented in the tables of the results. Where data was not available for all patients, the n-value is specified in the results.

## Results

**Figure 1:**
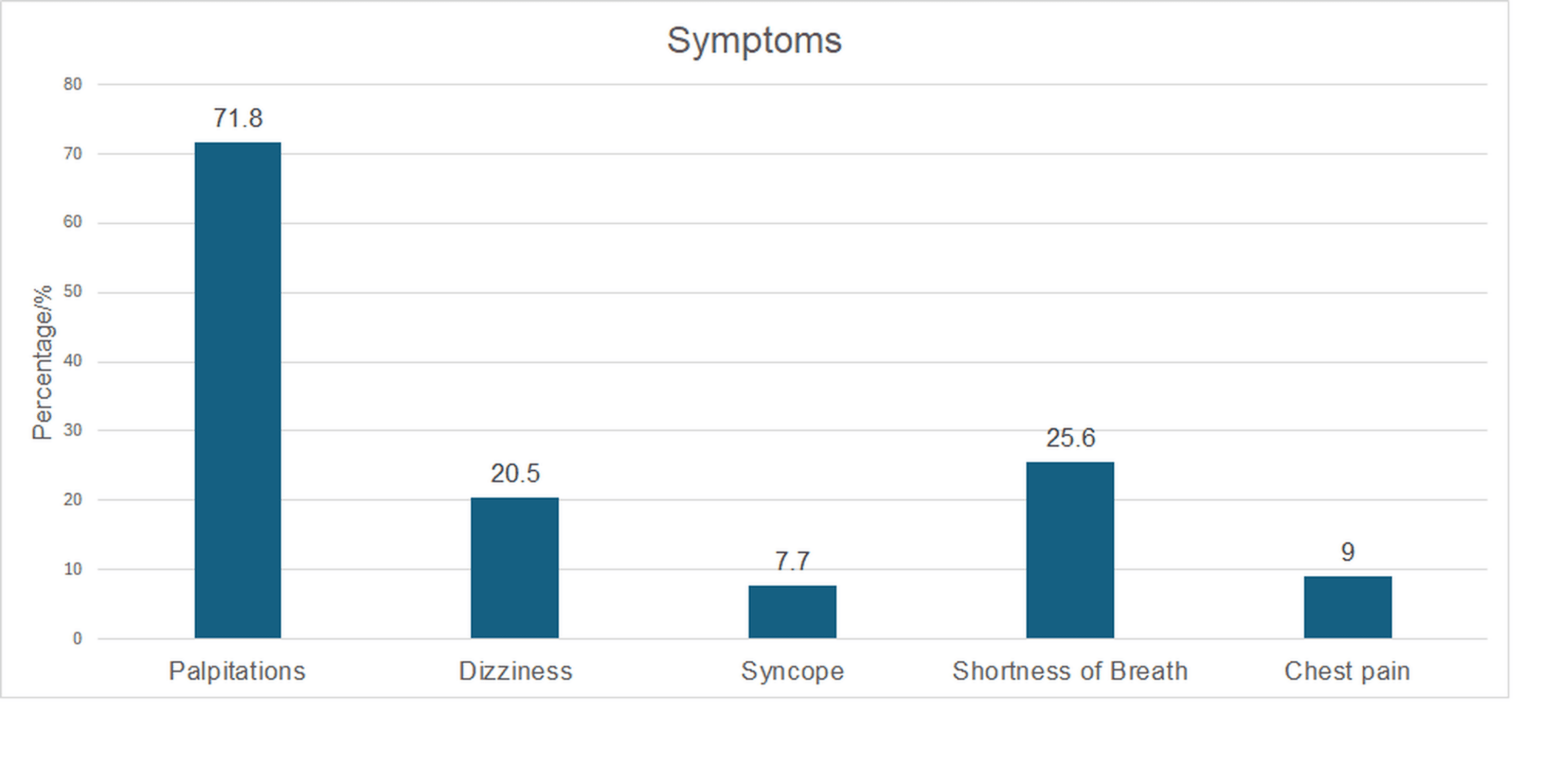
Common symptoms exhibited in symptomatic HCM patients HCM - hypertrophic cardiomyopathy

Thirty-nine patients were identified with HCM. During the study period, 30 were male and nine were female. Apical and asymmetrical septal HCM were the most common variants of HCM, composing 15 (38.5%) and 16 (41%) of the total HCM patients, respectively. The other types of HCM found were focal and concentric HCM. The median age at diagnosis was 47.46±12.27 years. Twenty (51.3%) patients were found to be asymptomatic. Figure 1 displays the common symptoms exhibited in HCM patients. Palpitations manifested in 28 (71.8%) patients with diagnosed HCM. Demographic characteristics are summarized in Table 2. Hypertension was seen in 29 (74.4%) patients, while diabetes mellitus manifested in 13 (33.3%) patients. A family history of HCM was present in four (10.3%), while a family history of SCD was found in nine (23.1%) patients. The presence of fibrosis was found in 21 (53.8%) patients. Of the 30 patients with HCM, 12 (57.1%) had severe fibrosis as compared to six (28.6%) and three (14.3%) patients with mild and moderate, respectively. 

**Table 2:** Characteristics of patients with HCM HCM - hypertrophic cardiomyopathy; IQR - interquartile range; SAM - systolic anterior motion of mitral valve ^a^ skewed to the left

Demographic characteristics	n (%)
Age, mean±SD	47.46±12.27
Sex	
Male	30 (76.9)
Female	9 (23.1)
Signs and symptoms	
Symptomatic	19 (48.7)
Asymptomatic	20 (51.3)
Murmurs	16 (41.0)
Age at diagnosis	
≤50	24 (61.5)
>50	15 (38.5)
Type of HCM	
Apical	15 (38.5)
Asymmetrical septal	16 (41.0)
Others	8 (20.5)
Diabetes mellitus	13 (33.3)
Hypertension	29 (74.4)
Family history of sudden cardiac death	9 (23.1)
Family history of hypertrophic cardiomyopathy	4 (10.3)
Implantable cardioverter defibrillator	5 (12.8)
Beta-blockers	28 (71.8)
Calcium channel blockers	18 (46.2)
Amiodarone	4 (10.3)
Left ventricular hypertrophy	30 (76.9)
ST-T changes	35 (89.7)
Presence of ventricular tachycardia (n=27)	5 (18.5)
Presence of myocardial fibrosis (n=30)	21 (53.8)
Severity of myocardial fibrosis (n=21)	
Mild	6 (28.6)
Moderate	3 (14.3)
Severe	12 (57.1)
Presence of obstruction (n=38)	7 (18.4)
Presence of SAM (n=38)	8 (20.5)
Presence of mitral regurgitation (n=38)	30 (78.9)
Sudden death risk score (n=36)	
<4	28 (77.8)
4 – 6	6 (16.7)
>6	2 (5.6)
Median risk score (n=37), median (IQR)	1.79 (1.93)^a^

Some significant differences were noted when comparing apical HCM and asymmetrical septal HCM, as seen in Table 3. Asymmetrical septal HCM patients had higher rates of murmurs compared to apical HCM (56.3% vs 6.7%, p=0.001). Fibrosis was more predominant in apical HCM compared to asymmetrical septal HCM (83.3% vs 60%, p=0.032). The median sudden death risk score between apical HCM and asymmetrical septal HCM was significantly different (p=0.025), 1.44 (IRQ: 0.85) and 2.05 (IRQ: 1.78), respectively (Table 4). There was no significant difference (p=0.828) between median age at diagnosis in apical and asymmetrical septal HCM patients, 45 (IRQ: 14) and 49 (IRQ: 25.25), respectively. No other significant differences were observed between apical HCM and asymmetrical septal HCM patients. 

**Table 3:** Characteristics of apical and asymmetrical septal HCM patients SAM - systolic anterior motion of mitral valve; HCM - hypertrophic cardiomyopathy ^a^ Fisher's exact test; ^b^ n=13 of apical HCM patients; ^c^ n=15 of asymmetrical septal HCM patients; ^d^ n=12 of apical HCM patients; ^e^ n=9 of asymmetrical septal HCM patients​​​​​​​;^ f^ n=14 of apical HCM patients; ^g^ n=10 of apical HCM patients

Charachteristics	Apical HCM (n=15), n (%)	Asymmetrical septal HCM (n=16), n (%)	p-value^a^
Sex			
Male	13 (86.7)	11 (68.7)	0.399
Female	2 (13.3)	5 (31.3)	-
Age at diagnosis			
≤50	10 (66.7)	9 (56.3)	0.911
>50	5 (33.3)	7 (77.8)	-
Presence of symptoms	5 (41.7)	5 (33.3)	0.497
Murmurs	1 (6.7)	9 (56.3)	0.001
Diabetes mellitus	5 (41.7)	2 (13.3)	0.073
Hypertension	9 (75.0)	11 (73.3)	0.803
Family history of sudden cardiac death	2 (16.7)	4 (26.7)	0.49
Family history of hypertrophic cardiomyopathy	0 (0.0)	3 (20.0)	0.236
Implantable cardioverter defibrillator	2 (16.7)	2 (13.3)	1
Beta-blockers	9 (75.0)	12 (80.0)	0.9
Calcium channel blockers	5 (41.7)	7 (46.7)	1
Amiodarone	0 (0.0)	2 (13.3)	0.236
Left ventricular hypertrophy	8 (66.7)	11 (73.3)	0.79
ST-T wave changes	11 (91.7)	13 (86.7)	0.662
Presence of myocardial fibrosis ^c d^	10 (83.3)	9 (60.0)	0.032
Severity of myocardial fibrosis ^g e^			
Mild	4 (40.0)	9 (100.0)	0.44
Moderate	1 (10.0)	0 (0.0)	-
Severe	5 (50.0)	0 (0.0)	-
Presence of obstruction ^f^	2 (18.2)	1 (6.7)	0.371
Presence of SAM ^f^	1 (9.1)	2 (13.3)	0.064
Presence of mitral regurgitation ^f^	7 (63.6)	13 (86.7)	0.173
Sudden death risk score ^b c^			
<4	11 (84.6)	11 (73.3)	0.784
4 – 6	2 (16.4)	2 (13.3)	-
>6	0 (0.0)	2 (13.3)	-

**Table 4:** Median age and sudden death risk score of apical and asymmetrical septal HCM patients IQR - interquartile range; HCM - hypertrophic cardiomyopathy ^a^ Mann-Whitney test; ^b^ n=13; ^c^ skewed to the left

Variable	Apical HCM, median (IQR)	Asymmetrical septal HCM, median (IQR)	Z-statistic​​​​​^a^	p-value^a^
Median age at diagnosis	45.00 (14.00)	49.00 (25.25)	-0.218	0.828
Median sudden death risk score	1.44 (0.85)^b c^	2.05 (1.78)^c^	-2.237	0.025

Table 5 summarizes the clinical characteristics of male and female HCM patients.

**Table 5:** Clinical characteristics of male and female HCM patients SAM - systolic anterior motion of mitral valve ^a^ Fisher's exact test; ^b^ n=23 of male patients;^ c ^n=28 of male patients; ^d ^n=7 of female patients

Clinical characteristics	Male (n=29), n (%)	Female (n=10), n (%)	p-value ^a^
Presence of symptoms (symptomatic)	12 (41.4)	7 (70.0)	0.155
Murmurs	10 (34.5)	6 (60.0)	0.264
Diabetes mellitus	8 (27.6)	4 (40.0)	0.253
Hypertension	21 (72.4)	7 (70.0)	1.000
Family history of sudden cardiac death	6 (20.7)	2 (20.0)	0.669
Family history of hypertrophic cardiomyopathy	2 (6.9)	2 (20.0)	0.267
Implantable cardioverter defibrillator	4 (13.8)	1 (10.0)	1.000
Beta-blockers	22 (75.9)	5 (50.0)	0.424
Calcium channel blockers	12 (41.4)	5 (50.0)	0.465
Amiodarone	2 (6.9)	2 (20.0)	0.267
Left ventricular hypertrophy	23 (79.3)	6 (60.0)	0.669
ST-T changes	27 (93.1)	7 (70.0)	0.267
Presence of myocardial fibrosis ^b d^	16 (69.6)	5 (71.4)	0.889
Presence of obstruction	5 (17.9)	2 (20.0)	1.000
Presence of SAM	6 (20.7)	2 (20.0)	1.000
Presence of mitral regurgitation	21 (75.0)	9 (90.0)	0.653

There was no significant difference (p=0.116) in the median age at diagnosis for males and females, 45.50 (IRQ: 16.75) and 52 (IRQ: 15.50), respectively. The median sudden death risk score between males and females was not significantly different (p=0.723), 1.72 (IRQ: 1.95) and 2.17 (IRQ: 1.97), respectively (Table 6).

**Table 6:** Median age at diagnosis and sudden death risk score of HCM males and female patients IQR - interquartile range; HCM - hypertrophic cardiomyopathy ^b^ Mann-Whitney test; ^b ^skewed to the left

Variable	Male, median (IQR)	Female, median (IQR)	Z-statistic ^a^	p-value ^a^
Median age at diagnosis	45.50 (16.75)	52.00 (18.50)	-1.384	0.116
Median sudden death risk score	1.72 (1.95) ^b^	2.17 (1.97) ^b^	-0.354	0.723

There was no significant difference between myocardial fibrosis and median sudden death risk score (p=0.143) as seen in Table 7.

**Table 7:** Median sudden death risk score and myocardial fibrosis of HCM patients HCM - hypertrophic cardiomyopathy ^a^ n=21; ^b^ Mann-Whitney test

Variable	Presence of fibrosis^a^			p-value^b^
	Yes		No	
Median sudden death risk score	1.57 (0.90)	2.85 (3.78)		0.143

## Discussion

There were differences in the demographics of HCM patients in Brunei Darussalam compared to other countries. The number of patients with apical and asymmetrical septal HCM was almost the same. In Qatar patients, asymmetrical septal HCM was more prevalent than apical HCM [7]. In contrast, apical HCM is more common in Asian countries than in Western countries [17]. The presence of asymptomatic patients, 51.3%, in our study is generally higher compared to studies done in Japan (37%) and Qatar (23.7%) [7,18]. Our study showed that the presence of murmurs was more common in HCM patients compared to the Qatar study [7]. We observed that 21 (53.8%) of the HCM patients had myocardial fibrosis. This is lower than expected compared to other studies where fibrosis is found in up to about 80% of HCM patients [19-20]. However, in our study, we found that apical HCM had a similar incidence of fibrosis (83.3%).

Some differences were noted among apical HCM and asymmetrical septal HCM patients. The presence of murmurs was significantly higher in asymmetrical septal HCM compared to apical HCM patients (p=0.001). It was also noted that the presence of fibrosis is higher in apical HCM compared to asymmetrical septal HCM patients (p=0.032). However, there were no significant differences in the severity of fibrosis (p=0.440). There was no significant difference between the prevalence of apical HCM and asymmetrical septal HCM in both genders. It is reported that a family history of HCM was more common in asymmetrical septal hypertrophy compared to apical HCM [21]. In our study, there was no significant difference between apical HCM, 0 (0%), and asymmetrical septal HCM, 3 (20%), in terms of family history of HCM (p=0.236). Family history of SCD was also not significantly different (p=0.490) between apical HCM, 2 (16.7%), and asymmetrical septal HCM, 4 (26.7%). However, this might simply occur due to a small sample size.

There was no significant difference in the age at diagnosis between apical HCM and asymmetrical septal HCM patients. However, asymmetrical septal HCM patients were found to have a higher median sudden death risk score compared to apical HCM patients (p=0.025). This is similar to a study done in Korea where asymmetrical septal HCM was shown to have a poorer prognosis compared to apical HCM [22]. The poorer prognosis compared to apical HCM may be attributed to the higher occurrence of myocardial fibrosis in asymmetrical septal HCM patients, which warrants future studies. There was no significant difference in the usage of automated implantable cardioverter defibrillator (AICD) as treatment. This may be due to the small sample size and presence of outliers.

Similar studies were conducted in Taiwan, Qatar, and New York, which demonstrated that the prevalence of HCM was found to be higher in males compared to females [7,10,23]. The median age at diagnosis was similar to studies in Qatar and New York but lower than in Japan [7,18,23]. The median age at diagnosis in males was found to be significantly lower or slightly lower than in females [7,10,18,23]. The median age at diagnosis for males was slightly lower than that of females, but it was not significantly different in our study. Inconsistency with the findings of other studies might be due to the small sample size. Also, the possible explanation of why males were diagnosed at a younger age may be due to the difference in the probability of symptom manifestation, where symptoms tend to manifest earlier in males than females, and thus cause the delay in diagnosis of HCM in females. Another possible reasoning might be due to clinician bias, as males get more attention because cardiac diseases are more common in males than females [18].

Generally, female HCM patients were more likely to manifest symptoms than males in our study. There was a slight difference in the presence of murmurs between males and females, which corresponds with a study by Kubo et al. [18]. However, there was no statistical difference between presentations of symptoms in males and females, 41.4% and 70%, respectively, despite females showing a slightly higher occurrence of symptoms compared to males. The reason for this difference is unclear, but most likely it is due to small sample size and delay of diagnosis until symptoms are more severe or noticeable [18]. In our study, the presence of systolic anterior motion of the mitral valve was low and insignificantly different in both genders. In HCM, the presence of systolic anterior motion of the mitral valve tends to cause obstruction in patients. This, in turn, manifests as symptoms in HCM patients [24,25]. Therefore, the low presentation of systolic anterior motion of the mitral valve may explain the low presentation of symptoms and obstruction in our study. There were no significant differences between genders regarding the presence of myocardial fibrosis and its severity. It is also worth mentioning that of the 30 patients with hypertrophy, nine patients did not have findings of fibrosis. 

According to Elliot et al. [12], a low risk score of less than 4%, the use of AICD is generally not indicated.Use of AICD may or should be considered if the sudden cardiac death risk score is above 4%. In our findings, there was no significant difference between the genders in terms of the sudden cardiac death risk score. The median sudden cardiac death risk score for males and females was 1.72% (IRQ: 1.95) and 2.17% (IRQ: 1.97), which is less than 4%. This corresponds to the lower usage of AICDs as part of the treatment regime.

The patients without findings of fibrosis were not investigated for infiltrative diseases such as cardiac amyloid, which can be potentially looked at in future studies, as they can mimic HCM [26]. Further recommendations for future studies could include a comparison of symptoms between both genders, potential clinical characteristics that could affect prognosis, and investigating whether the severity of fibrosis correlates with the increased risk score of sudden cardiac death.

There are limitations in our study. Some patient records or information may be missing or not be up to date, as BruHIMS was only implemented in 2012, and the HCM clinic was started in 2011. In addition, a historic fire incident at the department contributed to some missing information during data collection. Since HCM may manifest with subtle symptoms or asymptomatically, patients are unaware of the underlying disease. Patients are diagnosed incidentally in general screening assessments by ECG, i.e., suspected HCM from ECGs from general practitioner (GP) clinics or emergency department admissions for another medical condition. Therefore, there may be an underestimation of the number of HCM patients in Brunei Darussalam. Due to the small population size, only a small sample size was available for analysis. This restricts the power of analytical tests performed. Bonferroni correction was not applied due to the small number of comparisons and given nature of the hypothesis, the increased risk of type 1 error is considered acceptable. There is also a potential for selection bias due to single reliance on a single HCM clinic. Despite the identified limitations, the data represents a true picture of HCM in Brunei Darussalam.

## Conclusions

In conclusion, asymmetrical septal HCM has a generally poorer prognosis compared to apical HCM in view of the increased sudden cardiac death risk score in this study. A high index of suspicion, screening opportunities, early diagnosis, availability of diagnostic resources, and a national screening protocol are necessary to represent a true picture of HCM in Brunei Darussalam.
